# Intracranial Disease and Choked Disc

**Published:** 1882-06

**Authors:** 


### INTRACRANIAL DISEASE AND CHOKED DISC.

Dr. Edward G. Loring (*New York Medical Journal and Obstetrical Review*) gives the following conclusions: 1. That the vaso-motor theory, as advanced by Benedikt, is not sufficient to explain either the mode of transmission of the morbid iritation within the head, or the resulting neuritis optica. 2. That the irritation is conveyed, not by the isolated fibres of the sympathetic system, as stated by Benedikt, but through the agency of the trigeminus. 3. That choked disc or papillitis, in connection with brain disease, is the expression of an irritation or compression of certain intracranial fibres of the fifth pair which preside over the blood-supply of the disc and neighboring parts, and also maintain the healthy processes of waste and repair of the tissues themselves. This being so, he adds, the same analogies and distinctions between "irritation" and "inflammation" can be made here as with sympathetic ophthalmia, so that here, as well as there, the irritation may exist as such for an indefinite time, or may so reduce the vitality and resisting power of the tissue of the disc and surrounding parts as to develop gradually, or explode suddenly, into an actual inflammation—that is, into a neuritis. The immediate and exciting cause of this neuritis may then be either an external one, such as exposure to cold or heat, over-exertion, either mental or physical, or, indeed, too much exposure to light, the effects of which, under the weakened condition of the organ, may be looked upon as a "traumatism;" or the exciting cause may be an internal one, such as some irritation from the condition of the blood and circulating fluids, either chemical or mechanical, either local or general, which, insufficient in itself to produce any bad effect upon a normal disc, may yet be just sufficient to produce a condition of inflammation in a part that is weakened and irritable.

